# Supplementary material for: Identification of a master transcription factor and a regulatory mechanism for desiccation tolerance in the anhydrobiotic cell line Pv11
Source: PLoS One. 2020 Mar 19;15(3):e0230218. doi: 10.1371/journal.pone.0230218 (PMC7082025; doi:10.1371/journal.pone.0230218)
Supplement: S1 Text — (PDF) [file pone.0230218.s008.pdf]

S1 Text

Weighted gene coexpression network analysis for time-series expression data for differentially expressed genes (other than genes for transcription factors) detected 27 coexpressed gene modules. The “gene-battery” hypothesis is that coexpressed genes have similar biological functions [1]. We performed gene ontology (GO) enrichment analysis to detect such function for each module (S3 Data).

Thioredoxin is encoded by an anhydrobiosis-related gene and is important in removing harmful reactive oxygen species (ROS) and protecting cells from ROS-induced damage through thiol-disulfide exchange that involves cysteine residues [2]. GO:0015035 (protein disulfide oxidoreductase activity) is related to this reaction. This GO was detected as enriched in the Dark red, Green, Green yellow, and Grey60 modules (groups of coexpressed genes) (p-values = 5.04×10^{-3}, 0.0295, 1.96×10^{-3}, and 5.78×10^{-4}, respectively).

Protein-L-isoaspartate (D-aspartate) O-methyltransferases (PIMTs) repair isomerized aspartic acid and are important in desiccation tolerance [3]. GO:0004719 (protein-L-isoaspartate (D-aspartate) O-methyltransferase activity) is related to this function. This GO was enriched in the Dark red and Grey60 modules (p-value = 8.38×10^{-12} and 1.38×10^{-3}, respectively).

Trehalose is crucial for desiccation tolerance in *P. vanderplanki* because it stabilizes the phospholipid membrane [4]. GO:00052992 (trehalose biosynthetic process) and GO:0015927 (trehalase activity) are related to the synthesis and degradation of this metabolite, respectively. These GOs were enriched in the Grey60 and Cyan modules, respectively (p-value = 0.0271, 0.0163).

DNA repair is important after desiccation [5]. GO:0006281 (DNA repair) is related to this function. This GO was enriched in the Red module (p-value = 1.68×10^{-3}).

Thus, GOs related to various functions previously reported as important in desiccation tolerance were enriched in various modules. On the other hand, the GOs related to LEA proteins, hemoglobin, or aquaporin were not detected in our analysis. For LEA, there is no registered GO. Only 2 differentially expressed genes for hemoglobin and aquaporin each were detected (S3 Data), whereas GO enrichment analysis detected many genes with the target GO in the same cluster.

References

1. Davidson EH. Genomic regulatory systems: in development and evolution. Elsevier; 2001.

2. Meyer Y, Siala W, Bashandy T, Riondet C, Vignols F, Reichheld JP. Glutaredoxins and thioredoxins in plants. Biochim Biophys Acta Mol Cell Res. 2008; 1783(4): 589–600.

3. Khare S, Linster CL, Clarke SG. The interplay between protein L-isoaspartyl methyltransferase activity and insulin-like signaling to extend lifespan in Caenorhabditis elegans. PLoS ONE. 2011; 6(6): e20850.

4. Clegg JS. Cryptobiosis—a peculiar state of biological organization. Comp Biochem Physiol B, Biochem Mol Biol. 2001; 128(4): 613–624.

5. Yamada TG, Suetsugu Y, Deviatiiarov R, Gusev O, Cornette R, Nesmelov A, et al. Transcriptome analysis of the anhydrobiotic cell line Pv11 infers the mechanism of desiccation tolerance and recovery. Sci Rep. 2018; 8.
