# Supplementary figures and images for: Identification of a master transcription factor and a regulatory mechanism for desiccation tolerance in the anhydrobiotic cell line Pv11
Source: PLoS One. 2020 Mar 19;15(3):e0230218. doi: 10.1371/journal.pone.0230218 (PMC7082025; doi:10.1371/journal.pone.0230218)

Mapping Rate

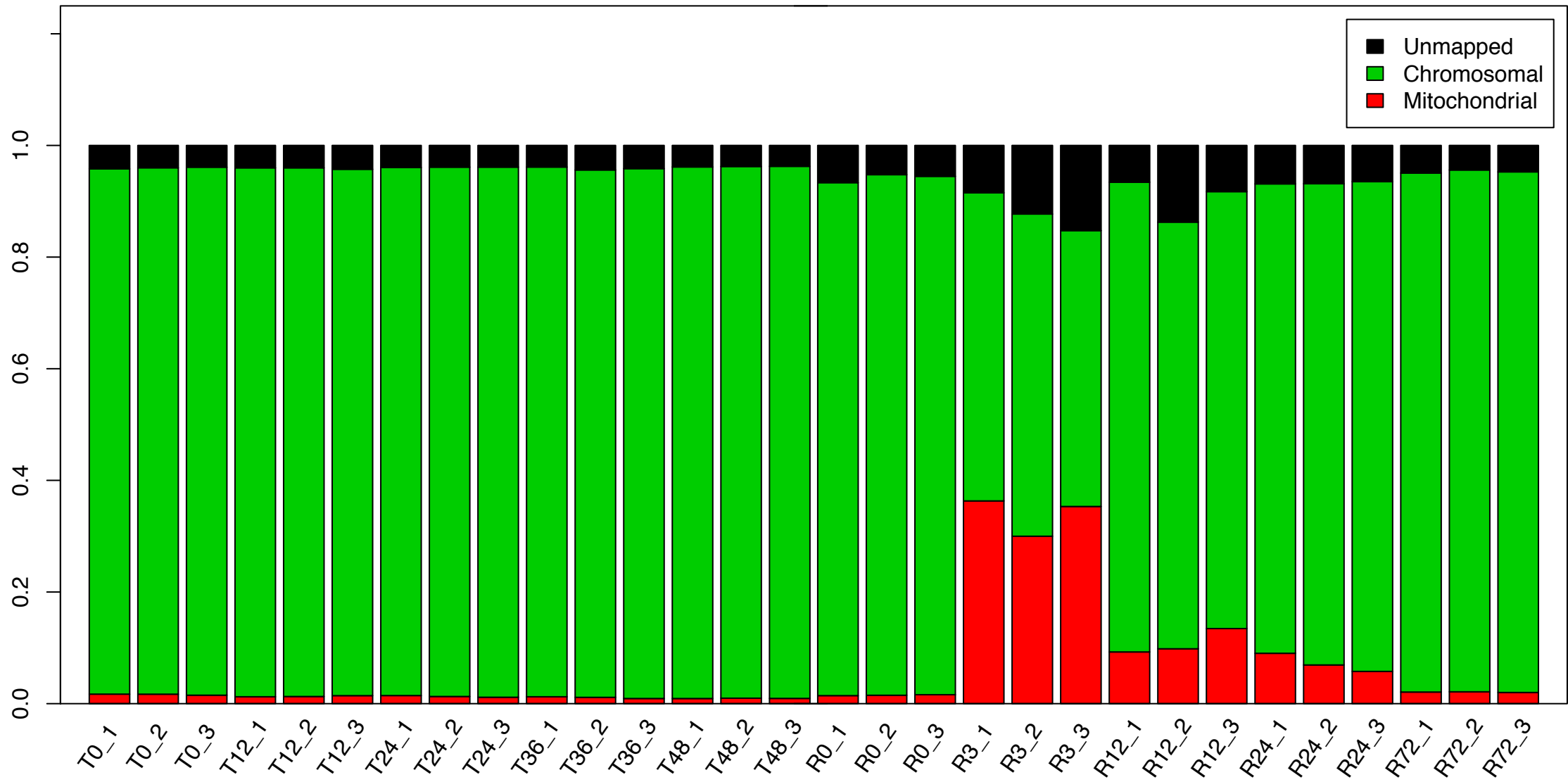

Supplement: S1 Fig — Three biological replicates per sample are shown. The mapping rate to the mitochondrial genome increased sharply just after rehydration. (PDF) [file pone.0230218.s009.pdf]

T0

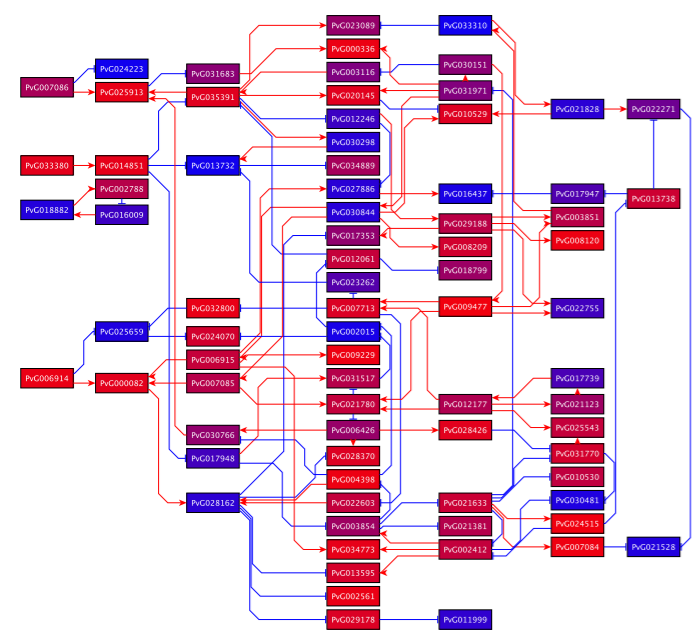

T12

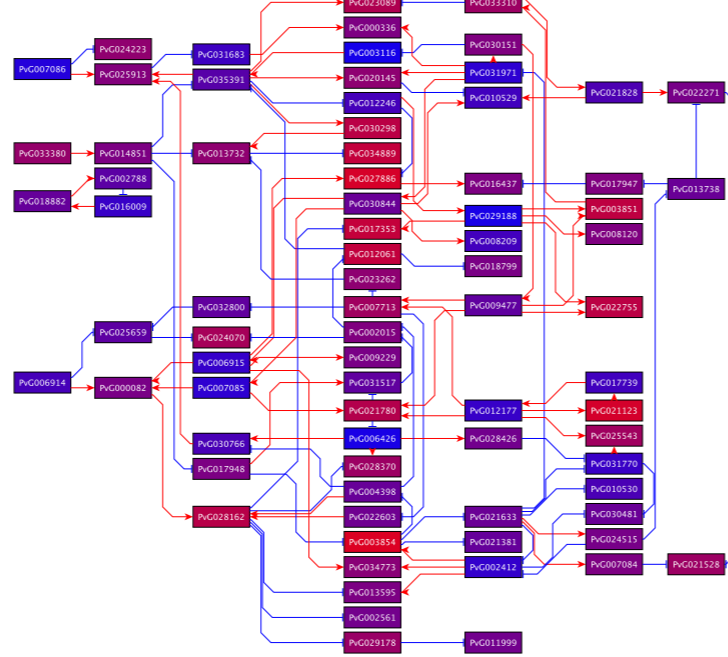

T24

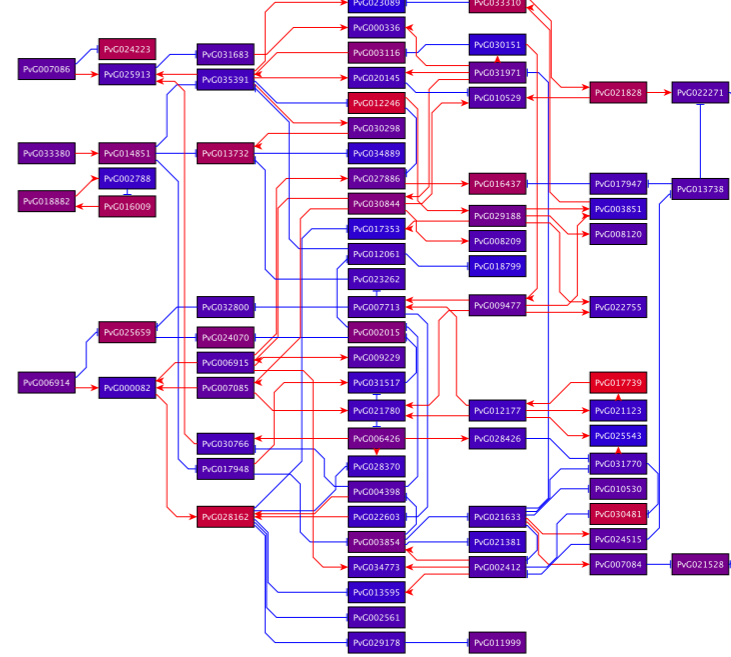

2.0

Z-Score

-2.0

T36

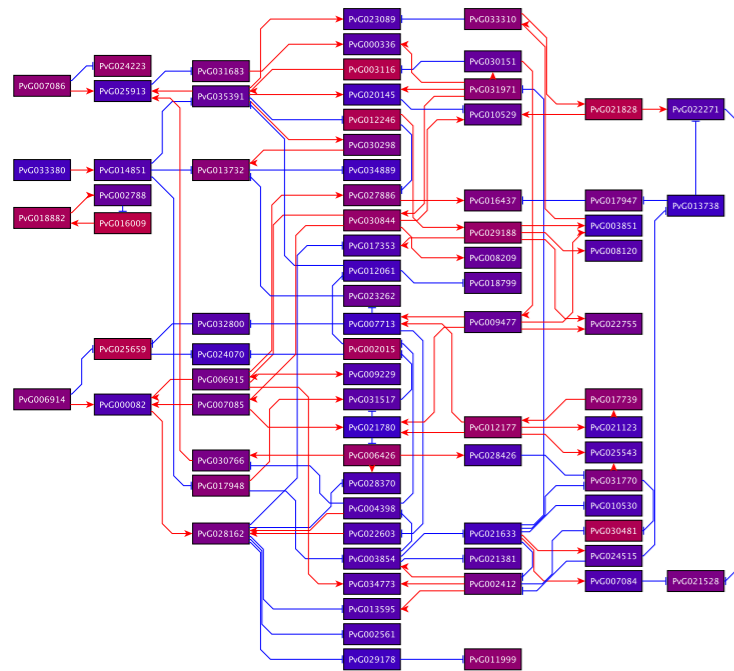

T48

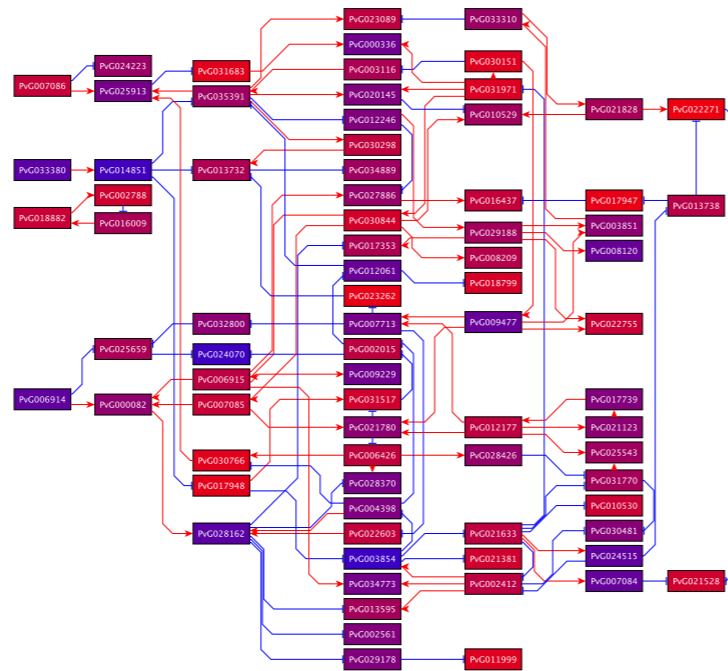

Supplement: S2 Fig — Each rectangular node refers to a transcription factor; transcription factor IDs are as defined in [19]; arrows show the inferred regulatory relationships (red, positive; blue, negative). The node color was based on the value of the mean Z-score in each sample calculated at each time point as reads per kilobase of exon per million mapped reads (RPKM) for each gene: red, high Z-score; blue, low Z-score. (PDF) [file pone.0230218.s010.pdf]

# R0

# R3

# R12

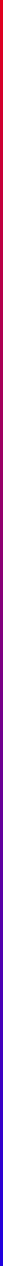

**-2.0**

# R24

# R72

Supplement: S3 Fig — Designations are as in S2 Fig. (PDF) [file pone.0230218.s011.pdf]

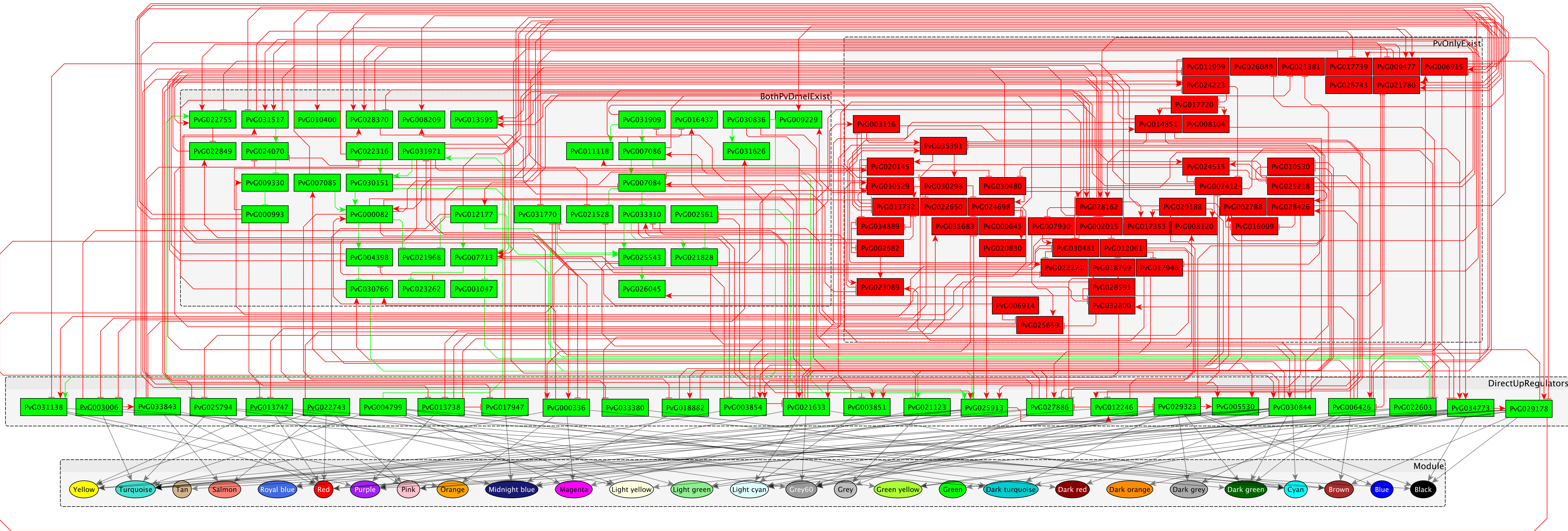

Supplement: S4 Fig — Rectangular nodes, transcription factors; circular nodes, modules. Transcription factors with sequence similarity to those in the D. melanogaster transcriptional regulatory network are shown in green (blastp, e-value < 1.0e-15); other transcription factors are shown in red. Regulatory relationships confirmed in both P. vanderplanki and D. melanogaster are shown as green arrows, and those specific for P. vanderplanki are shown as red arrows. Regulatory relationships between transcription factors and modules are shown as grey arrows (CLOVER, p-value < 0.05 and Granger causality test, adjusted p-value < 0.05, Benjamini-Hochberg method). Thus, the candidate regulatory network specific to desiccation tolerance is represented by red nodes and arrows. (PDF) [file pone.0230218.s012.pdf]

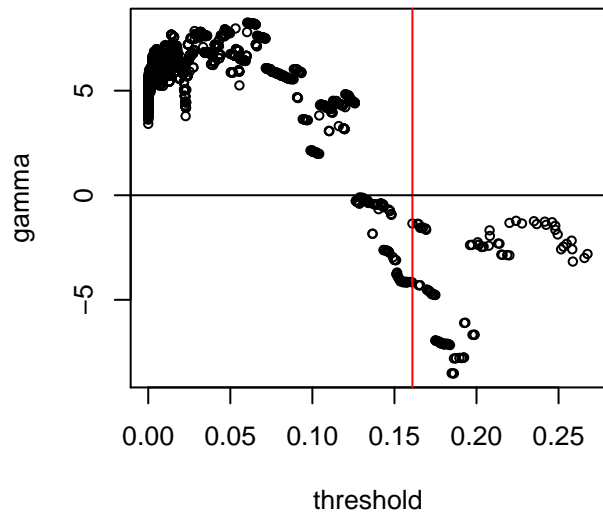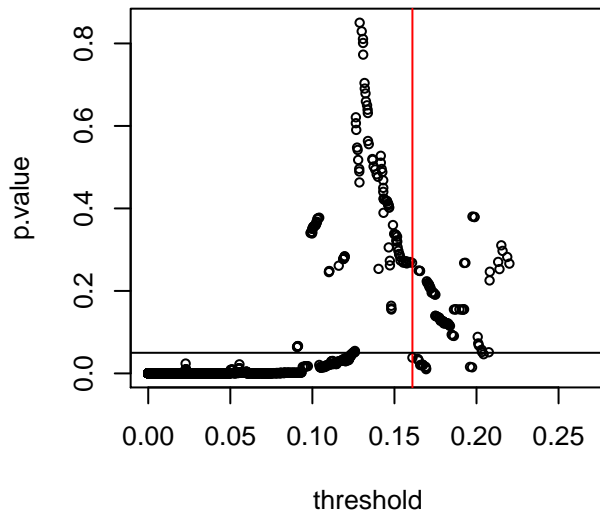

**$\gamma = -1.351$  , r.squared = 0.5386**  
**th = 0.160963**

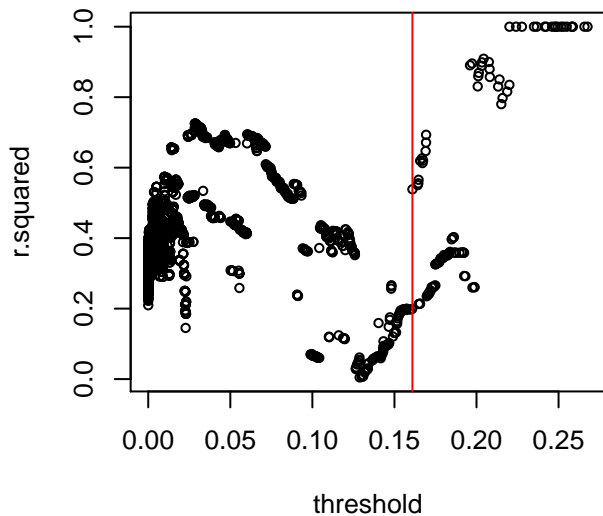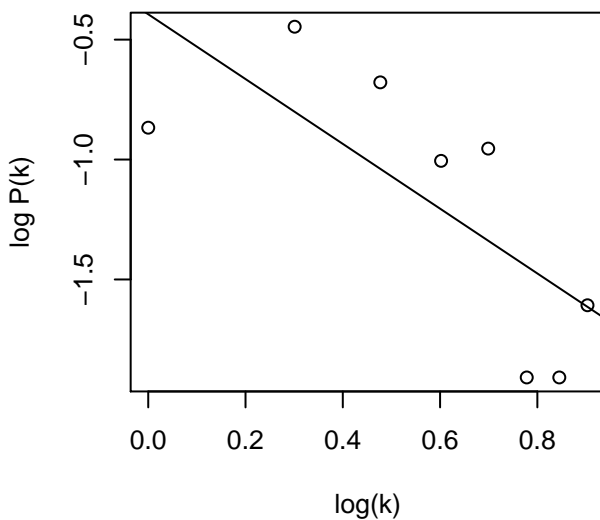

Supplement: S5 Fig — (upper left) The value of γ, which is the property of scale-free topology. (upper right) p-value for γ in F-test. (lower left) R2 values obtained by fitting linear relationships between degree (the number of regulations of the transcription factor) and degree distribution. Red lines show the minimum value of the threshold (0.160963) at which γ was negative and p-value was less than 0.05. (lower right) A plot of the log-log transformed degree distribution based on threshold = 0.160963. The line shows fitting result for the scale-free equation. (PDF) [file pone.0230218.s013.pdf]

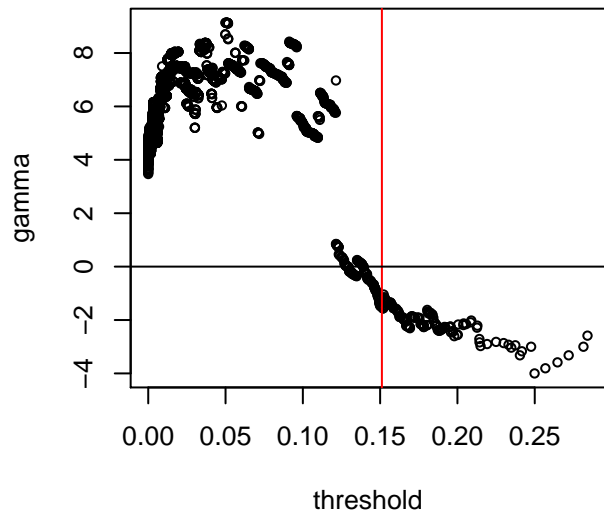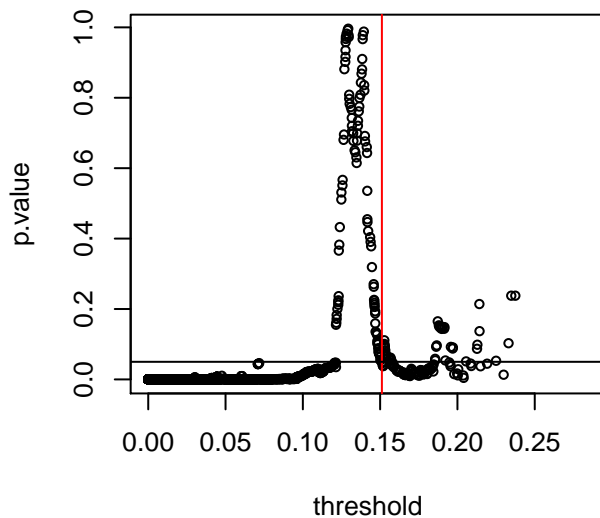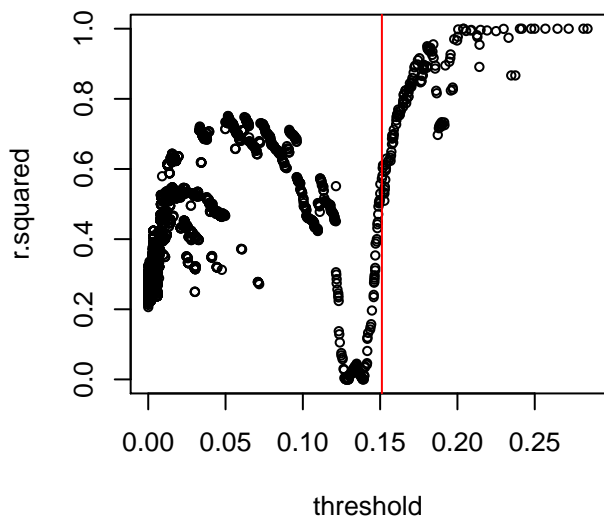

**$\gamma = -1.504$  ,  $r.squared = 0.5726$   
 $th = 0.151133$**

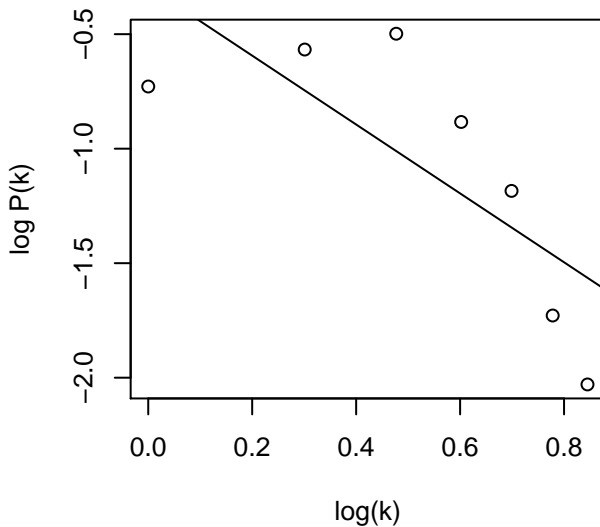

Supplement: S6 Fig — For panel descriptions and designations, see S5 Fig. The threshold value was 0.151133. (PDF) [file pone.0230218.s014.pdf]

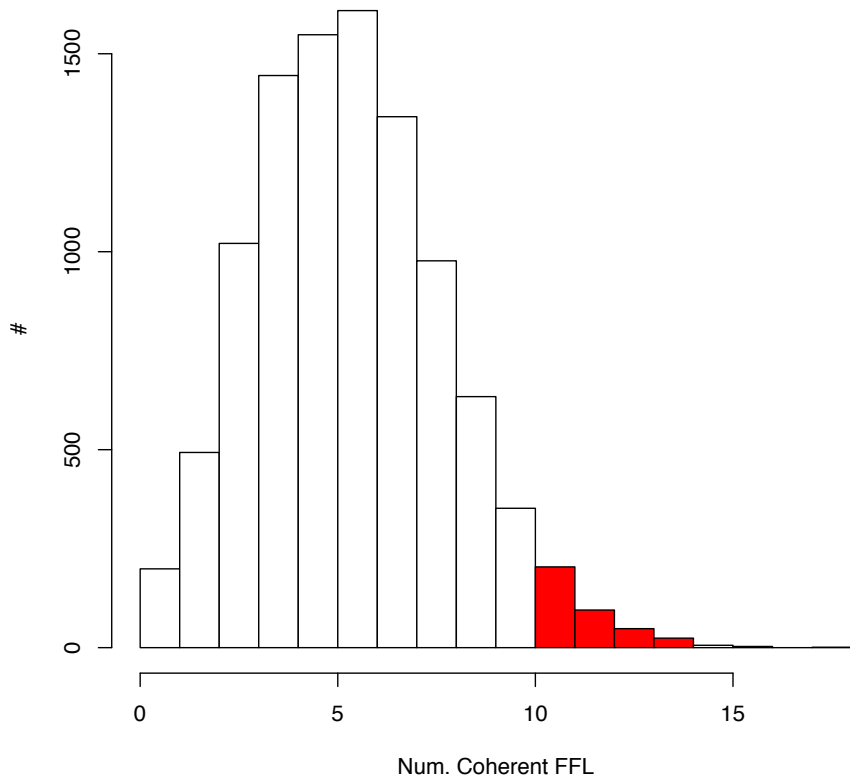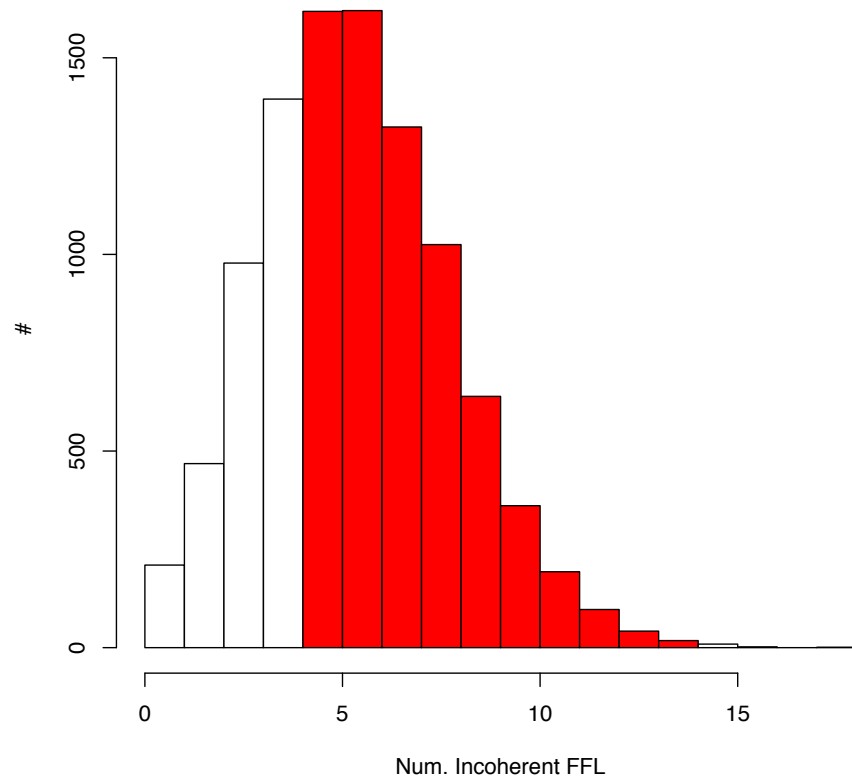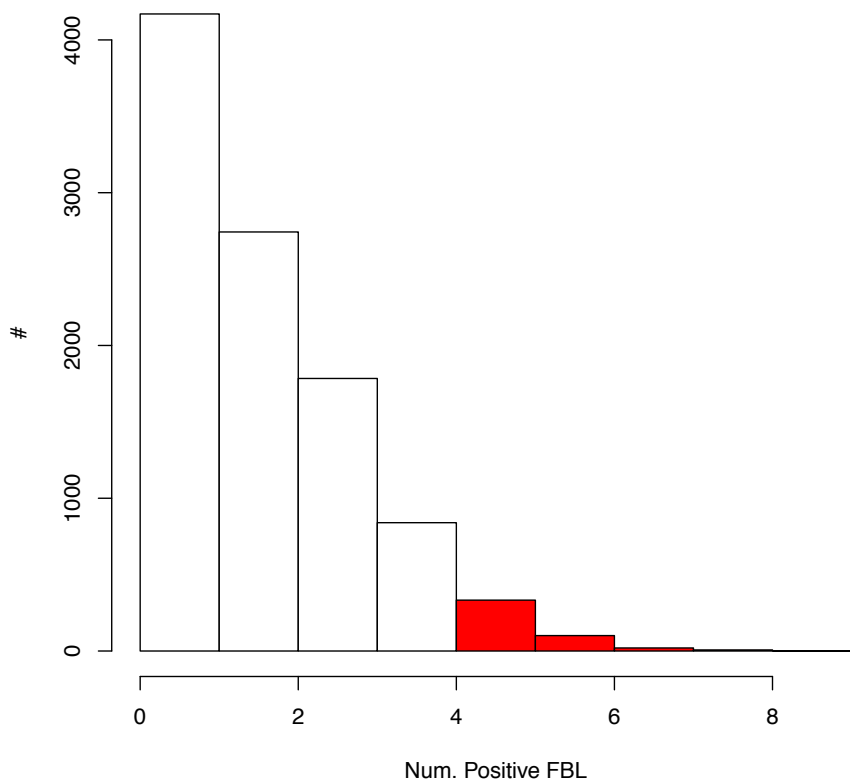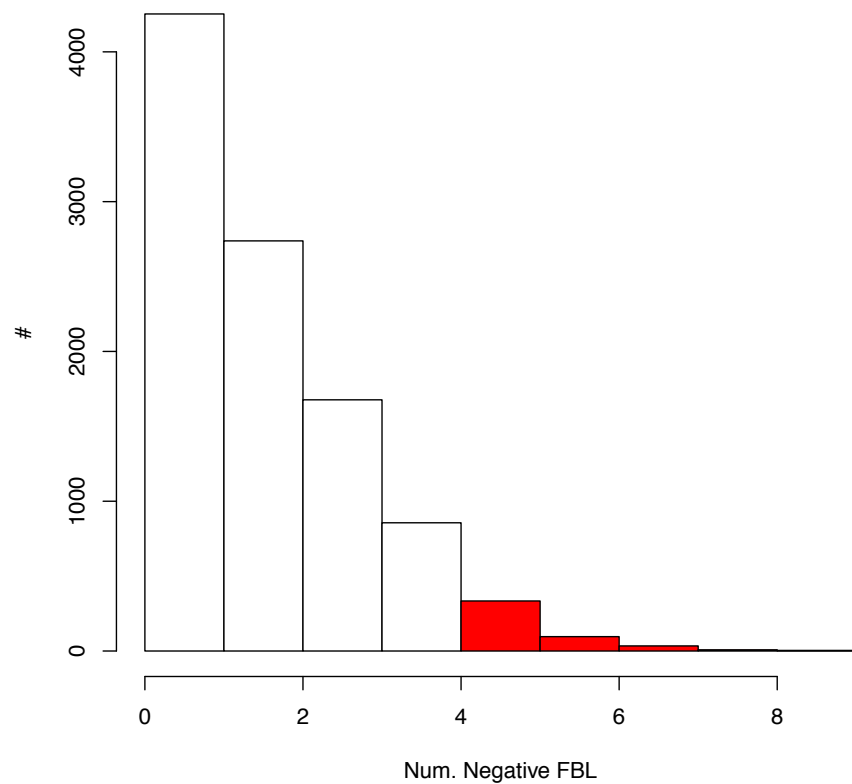

Supplement: S7 Fig — Histograms show the number of networks generated by the Erdös–Renyi model (#) categorized against the number of specific structures in the network. Red columns show the numbers of generated networks obtaining more than that of the inferred transcriptional regulatory network obtained by integration of those of trehalose pretreatment and rehydration. FFL, feed-forward loop; FBL, feedback loop. Rates of the red columns: coherent FFLs, 0.0177; incoherent FFLs, 0.5331; positive FBLs, 0.3087; negative FBLs, 0.3009. Thus, only the number of coherent FFLs was significant in the integrated network at the significance level of α = 0.05. (PDF) [file pone.0230218.s015.pdf]

## Scale independence

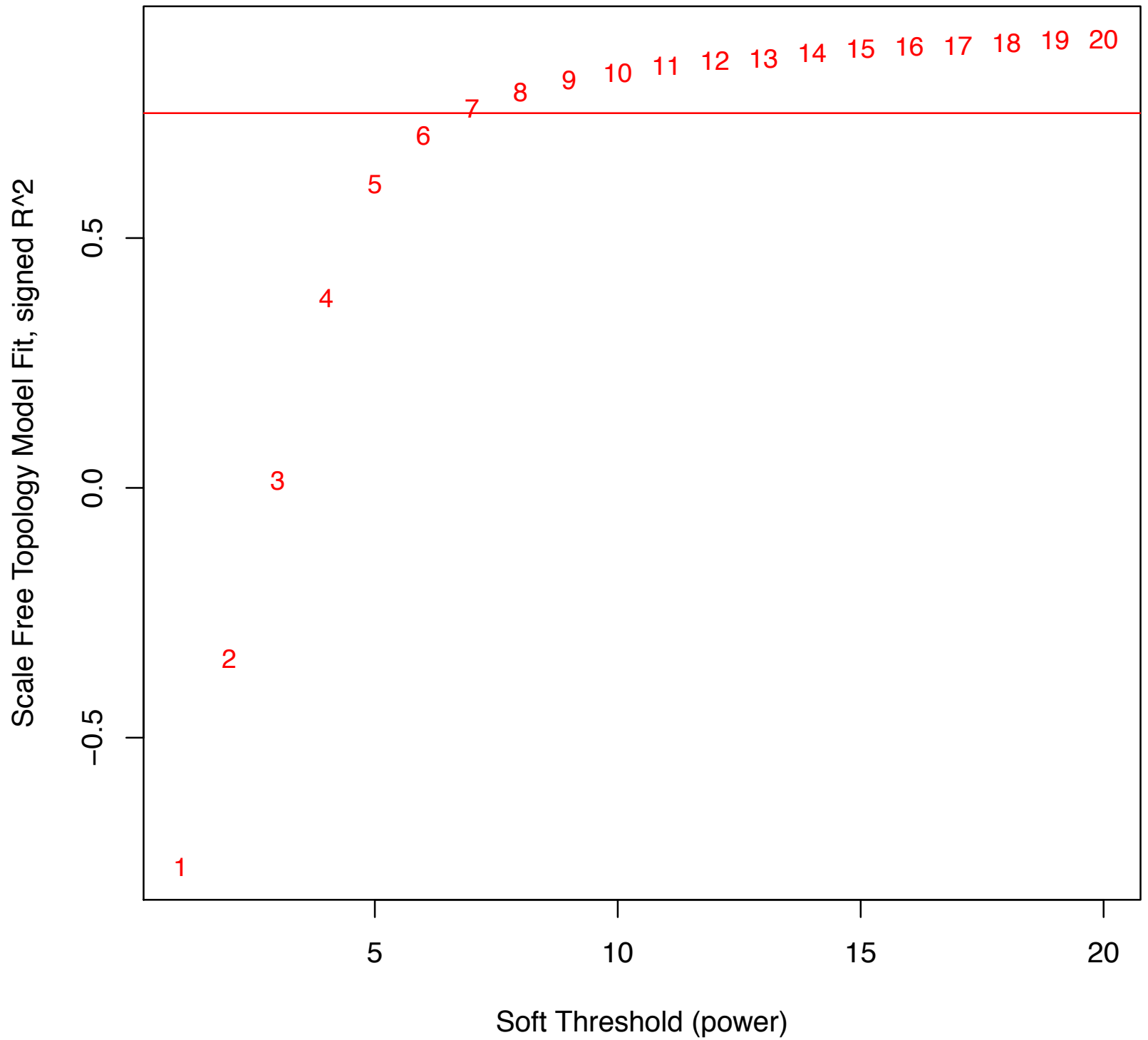

Supplement: S8 Fig — 7 of power satisfied that the signed R2 was more than 0.75 (red line). (PDF) [file pone.0230218.s016.pdf]

## Gene clustering on TOM-based dissimilarity

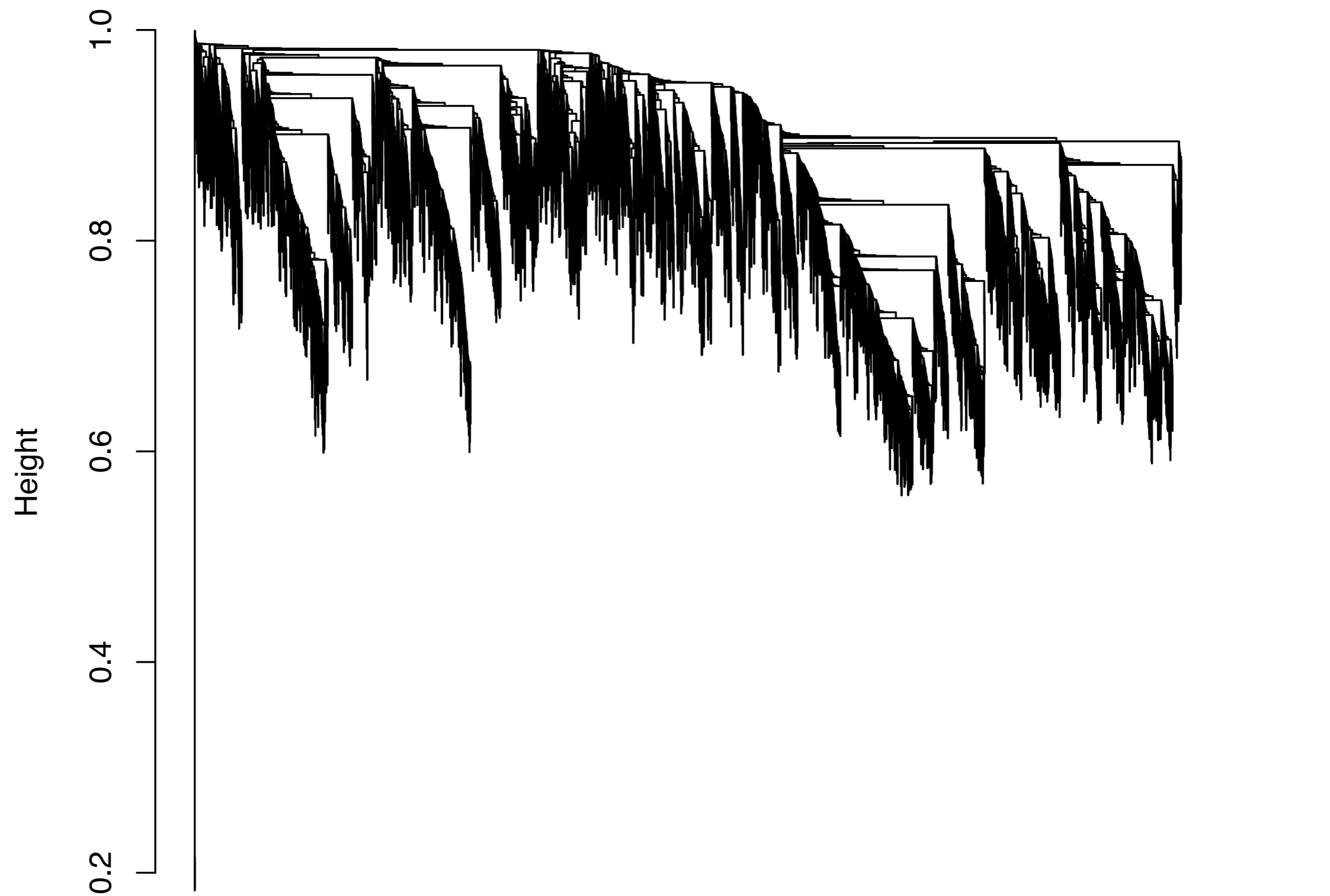

Supplement: S9 Fig — TOM was calculated from the Pearson correlation matrix of time-series Z-score for gene pairs raised by the estimated power of 7. (PDF) [file pone.0230218.s017.pdf]

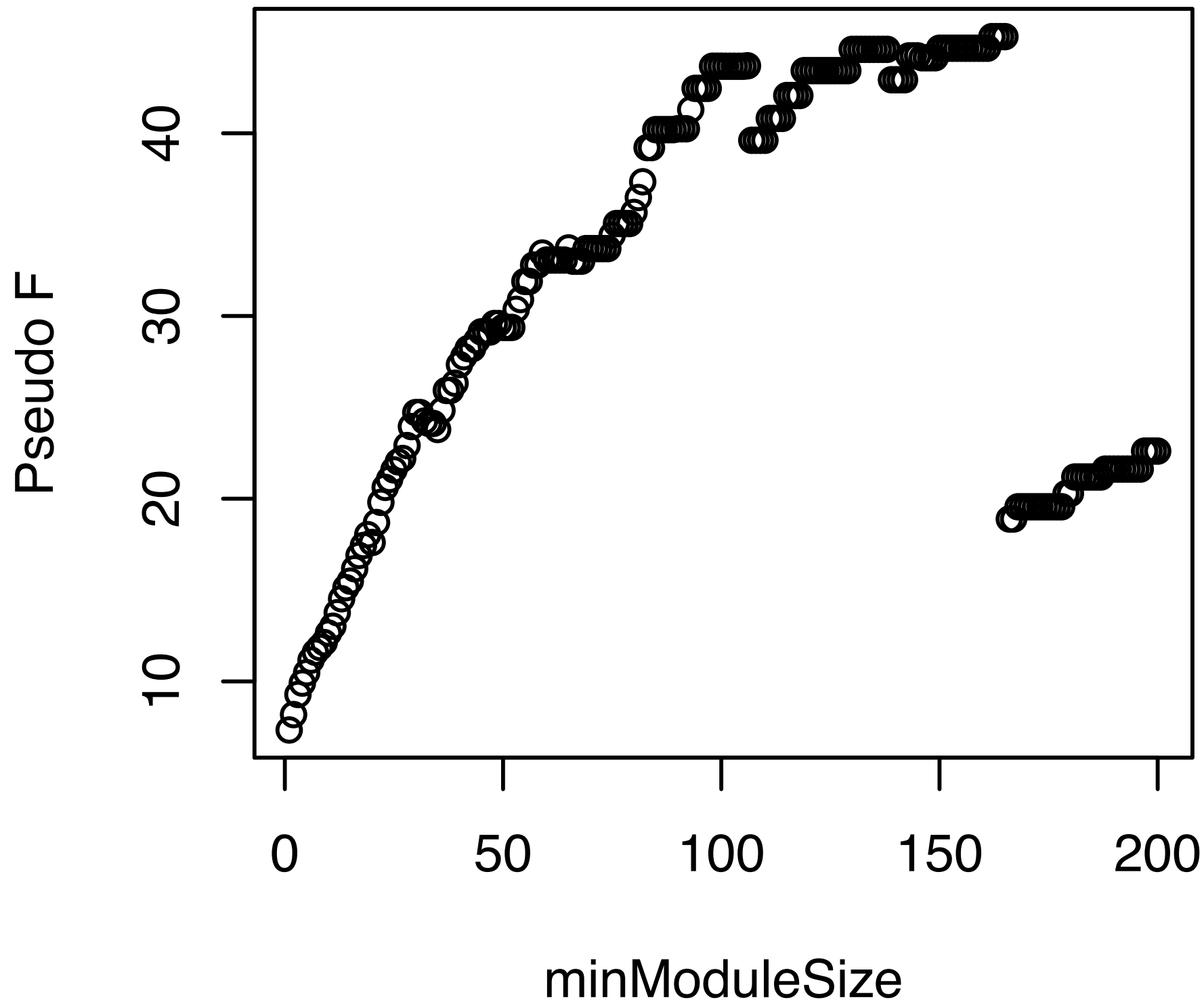

Supplement: S10 Fig — Pseudo-F increased from minModuleSize 1 to 165 and drastically decreased after 166; therefore, the minModuleSize was determined as 165. (PDF) [file pone.0230218.s018.pdf]
